# The association between infant and young child feeding practices and diarrhoea in Tanzanian children

**DOI:** 10.1186/s41182-018-0084-y

**Published:** 2018-01-30

**Authors:** Felix Akpojene Ogbo, Hillary Nguyen, Sabrina Naz, Kingsley E. Agho, Andrew Page

**Affiliations:** 1 Translational Health Research Institute, School of Medicine, Western Sydney University, Campbelltown Campus, Locked Bag 1797, Penrith, NSW 2571 Australia; 2 Prescot Specialist Medical Centre, Makurdi, Benue State Nigeria

**Keywords:** Breastfeeding, Diarrhoea, Complementary feeding, Children, Tanzania

## Abstract

**Background:**

Diarrhoea is a leading cause of child mortality in Tanzania. The association between optimal infant feeding practices and diarrhoea has been reported elsewhere, but the evidence has been limited to promote and advocate for strategic interventions in Tanzania. This study examined the association between infant and young child feeding (IYCF) practices and diarrhoea in Tanzanian children under 24 months.

**Methods:**

The study used the Tanzania Demographic and Health Survey data to estimate the prevalence of diarrhoea stratified by IYCF practices. Using multivariable logistic regression modelling that adjusted for confounding factors and cluster variability, the association between IYCF practices and diarrhoea among Tanzanian children was investigated.

**Results:**

Diarrhoea prevalence was lower in infants aged 0–5 months whose mothers engaged in exclusive breastfeeding (EBF) and predominant breastfeeding (PBF) compared to those who were not exclusively and predominantly breastfed. Infants aged 6–8 months who were introduced to complementary foods had a higher prevalence of diarrhoea compared to those who received no complementary foods, that is, infants who were exclusively breastfed at 6–8 months. Infants who were exclusively and predominantly breastfed were less likely to experience diarrhoea compared to those who were not exclusively and predominantly breastfed [adjusted odds ratio (AOR) 0.31, 95% confidence interval (CI) 0.16–0.59, *P* < 0.001 for EBF and AOR = 0.30, 95% CI 0.10–0.89, *P* = 0.031 for PBF]. In contrast, infants aged 6–8 months who were introduced to complementary foods were more likely to experience diarrhoea compared to those who received no complementary foods (AOR = 2.91, 95% CI 1.99–4.27, *P* < 0.001).

**Conclusions:**

The study suggests that EBF and PBF were protective against diarrhoeal illness in Tanzanian children, while the introduction of complementary foods was associated with the onset of diarrhoea. Strengthening IYCF (facility- and community-based) programmes would help to improve feeding behaviours of Tanzanian women and reduce diarrhoea burden in children under 2 years.

## Background

The early childhood period is considered one of the most important phases of life, determining the quality of health, behaviour and well-being across the lifespan [1]. The early year period also reflects a time of high vulnerability of the child to various adverse health outcomes such as diarrhoea [2]. In the past two decades, significant progress has been made in reducing diarrhoeal morbidity and mortality among children under 5 years old; however, recent reports have shown that diarrhoea is still a leading cause of under-five mortality in many developing countries [3, 4]. In sub-Saharan African (SSA) countries, including Tanzania, children are likely to die before their fifth birthday from illnesses such as diarrhoea and upper respiratory tract infection compared to their counterparts in developed countries [5–7]. Additionally, under-five deaths in developing countries are mainly attributable to preventable infectious diseases (including diarrhoea) in which appropriate breastfeeding, improved water and sanitation, routine vaccination programs and strong operational policies can play significant roles [8, 9].

Optimal breastfeeding practices protect children against diarrhoea-related diseases [10, 11], and mothers who optimally breastfeed their babies have been shown to have a reduced risk of type 2 diabetes mellitus and breast and ovarian cancers [5, 12]. A recent review indicated that only approximately 40% of infants aged 0–5 months in SSA countries were exclusively breastfed, reflecting gaps in optimal infant feeding practices in many communities [5]. The study also suggested that many infants were introduced to complementary foods too early in SSA countries. While the introduction of complementary foods to infants is crucial for infant growth and development, previous studies have indicated that the poor conditions in which those foods are prepared, stored or fed to the child as well as the immunological status of the child may contribute to the onset of diarrhoea [13–17]. Previous reports from Tanzania have indicated that the prevalence of exclusive breastfeeding (EBF) and continued breastfeeding at 1 year was high (50 and 92%, respectively) [18], and the median duration of any breastfeeding was 21 months, suggesting that some mothers exclusively breastfed for more than 6 months [19]. Nonetheless, the relationship between EBF and continued breastfeeding at 1 year and diarrhoea has not been investigated in Tanzania.

In 2016, the global burden of disease study indicated that diarrhoeal diseases contributed to the burden of under-five deaths and disabilities, which accounted for 5.4% of the total deaths and 5.9% of disability-adjusted life years in Tanzania [20, 21]. Additionally, the prevalence of diarrhoea among children under 5 years varied from 6% [22] to 22% [23] in Tanzania. Studies that investigated the impact of infant feeding practices on diarrhoea in SSA (e.g. Nigeria [10]) and Asia (e.g. Vietnam [24] and Bangladesh [7]) have been published, where EBF was protective against diarrhoeal illness. However, evidence on the association between infant and young child feeding (IYCF) practices and diarrhoea has been limited in Tanzania [25], where studies have primarily focused on HIV-exposed infants [26, 27].

A country-specific study that is focused on the relationship between IYCF practices and diarrhoea would be essential to public health experts to promote and advocate for strategic investments in IYCF programmes to improve child health outcomes in Tanzania. Additionally, public health programmes are largely funded by international agencies in Tanzania [28], and findings from this study will also be of interest internationally, particularly in the context of the renewed focus on child nutrition agenda at the global, regional and national levels [29]. The present study aimed to investigate the association between IYCF practices (i.e. EBF; early/timely initiation of breastfeeding; predominant breastfeeding; timely introduction of solid, semi-solid and soft foods and continued breastfeeding at 1 year) and diarrhoea in Tanzanian children under 24 months.

## Methods

### Data sources

The study used the Tanzania Demographic and Health Survey (TDHS, 2010) data, collected by the National Bureau of Statistics, Dar es Salaam, Tanzania, with technical assistance from the Inner City Fund (ICF) International, Maryland, USA. The TDHS collects maternal and child information that includes socio-demographics, child and female reproductive characteristics, obtained from a nationally representative sample of households. Using standardised face-to-face questionnaires, the TDHS collected information on IYCF practices from eligible women of childbearing age (15–49 years). For the IYCF practices, questions in the survey tool included information on breastfeeding and complementary feeding practices, as well as duration and diversity of the infant food provided. A weighted total sample of 10,139 eligible women were interviewed, yielding 96% response rate. Additional information on the data source (including sampling procedure and methodology for data collection) is described elsewhere [19, 30].

### Outcome variable

The study outcome was diarrhoea, defined as the passage of three or more loose or liquid stools per day, and was based on maternal recall of each child under 5 years of age in the household during the 2 weeks preceding the survey. Consistent with previous studies [18, 31], this analysis used information from the most recent live birth, aged less than 24 months, living with the respondent to reduce recall bias. Measurement of diarrhoea was based on the child age group for each IYCF practice [10].

### Exposure variables

The exposure variables for this study were the IYCF indicators, assessed based on the World Health Organization (WHO) definitions for assessing IYCF practices [32]:

- Early or timely initiation of breastfeeding: the proportion of children 0–23 months of age who were put to the breast within 1 h of birth.
- Exclusive breastfeeding: the proportion of infants 0–5 months of age who received breast milk as the only source of nourishment but allowed oral rehydration solution, drops or syrups of vitamins and medicines.
- Predominant breastfeeding: the proportion of infants 0–5 months of age who received breast milk as the main source of nourishment but allows water, water-based drinks, fruit juice, oral rehydration solution, drops or syrups of vitamins and medicines.
- Continued breastfeeding at 1 year: the proportion of children 12–15 months of age who were fed breast milk.
- Bottle feeding: the proportion of infants 0–23 months of age who received any liquid (including breast milk) or semi-solid food from a bottle with nipple/teat.
- Introduction of solid, semi-solid and soft foods: the proportion of infants 6–8 months of age who received solid, semi-solid or soft foods.

Early initiation of breastfeeding and EBF were incorporated into the analyses because of their association with reduced morbidity among children under 5 years [33, 34]. Predominant breastfeeding, bottle feeding, continued breastfeeding at 1 year and the introduction of solid, semi-solid and soft foods were included due to their effect on the increased risk of diarrhoeal morbidity and mortality among children under 5 years [10, 35–37].

A number of potential confounding variables were considered in the analyses based on previous studies [10, 24] and data availability. These variables were categorised as socioeconomic factors (mother’s employment, maternal education and household wealth), health service factor (frequency of antenatal care visit), individual factors (maternal age, child age and gender), and household factors (urban or rural, drinking water source and type of toilet facility). A detailed description of these variables is provided in Table 1. The household wealth index was calculated as a score of household assets (such as ownership of transportation devices, ownership of durable goods and household facilities), which was derived from a principal component analysis conducted by the National Bureau of Statistics, Dar es Salaam, Tanzania, and ICF International [19].

**Table 1:** Characteristics of the study population

Characteristics	*N*	% (95% confidence interval)
Socioeconomic		
Mother’s employment		
Not working	490	14.5 (12.7–16.4)
Working	2902	85.5 (83.6–87.3)
Mother’s education		
No schooling	865	25.5 (22.7–28.5)
Primary education	2278	67.2 (64.4–69.8)
Secondary and higher education	249	7.3 (6.1–8.8)
Household wealth		
Poor	1536	45.3 (41.7–49.0)
Middle	1360	40.1 (37.1–43.2)
Rich	496	14.6 (12.1–17.7)
Health Service		
Antenatal visits		
< 1	433	13.5 (12.0–15.3)
2–3	2280	71.5 (69.4–73.5)
4+	478	15 (13.2–17.0)
Individual		
Mother’s age (years)		
15–24	1281	37.8 (35.5–40.1)
25–34	1452	42.8 (40.8–44.9)
35–49	659	17.6 (17.6–21.4)
Sex of child		
Male	1708	50.4 (48.5–52.2)
Female	1684	49.6 (47.8–51.5)
Child age (months)		
0–3	562	17.3 (16.0–18.8)
4–5	860	26.5 (24.5–28.6)
6–11	865	26.6 (24.61–28.8)
12–17	139	4.3 (3.5–5.2)
18–23	821	25.3 (23.6–17.4)
Household		
Household location		
Urban	707	20.8 (16.7–25.7)
Rural	2685	79.2 (74.3–83.3)
Source of drinking water		
Not improved	5592	70.9 (67.0–74.6)
Improved	2288	29.0 (25.4–32.9)
Type of toilet		
Not improved	6697	84.9 (82.5–87.2)
Improved	1183	15.0 (12.8–17.5)

*N* weighted sample size

Source of drinking water was categorised as ‘Improved’ and ‘Not improved’. Improved drinking water source included residences where water was piped into the dwelling-yard, access to a public tap or standpipe, a tube well or borehole, protected well, protected spring, rainwater and/or bottled water. Not improved water source comprised access to an unprotected well, unprotected spring, tanker truck or cart with a drum, surface water, sachet water and/or another source [38].

Type of toilet was categorised as ‘Improved’ and Not improved’. Improved type of toilet included toilets such as flush or pour-flush toilets or piped to the sewer system, septic tank, and pit latrine; flush or pour-flush to septic tank; flush or pour flush to pit latrine; ventilated improved pit latrine; pit latrine with slab and/or compositing toilet. Not improved toilets comprised flush or pour-flush but not piped to sewer, septic tank or pit latrine; pit latrine without a slab or open pit; bucket or hanging toilet and no toilet facility or the use of bush or field [38].

### Statistical analysis

The exposures were expressed as dichotomous variables, where respondents (women aged 15–49 years) who exclusively breastfed were coded as ‘1’ and those who did not were coded as ‘0’. The same analytical approach was employed for other IYCF indicators. Preliminary analyses involved frequency tabulations of confounding variables (i.e. socioeconomic, health service, individual and household factors) in the TDHS, followed by an estimation of the prevalence of IYCF indicators, as well as a combination of IYCF practices and diarrhoea (and their corresponding 95% confidence intervals).

Univariable and multivariable logistic regression analyses were used to investigate the associations between IYCF practices and diarrhoea, adjusted for potential confounders. Regression models were restricted to the youngest living child aged < 24 months living with the respondent (women aged 15–49 years) to reduce recall bias. Prevalence estimates were calculated with the ‘svy’ function used to allow for adjustments for the cluster sampling design and regression modelling using the ‘xlogit’ function. All analyses were performed in Stata software version 14.0 (Stata Corporation, College Station, TX, USA).

### Ethics

Measure DHS/ICF International obtained ethical approval from the Medical Research Coordinating Committee (MRCC), the national health research coordinating body in Tanzania. All questionnaires used for the DHS were reviewed and approved by ICF International Institutional Review Board (IRB) to ensure they met the US Department of Health and Human Services regulations for the protection of human participants as well as the host country’s IRB, to ensure compliance with national laws. The datasets used are available to apply for online, and approval was obtained from Measure DHS/ICF International for the analysis.

## Results

Of the mothers surveyed (*N* = 10,139), 43.4% initiated breastfeeding within the first hour of birth and 48.6% exclusively breastfed for the first 6 months of their infants’ life (Table 2). Only 10.1% of infants aged 0–5 months were predominantly breastfed, while 3.7% were bottle-fed. Many infants aged 6–8 months were introduced to complementary foods (72.2%), while the majority of mothers continued breastfeeding at 1 year (89.6%).

**Table 2:** Prevalence of IYCF indicators and diarrhoea among children 0–23 months in Tanzania, TDHS 2010

	N*	Prevalence of IYCF indicators % (95% CI)^a^	Prevalence of diarrhoea % (95% CI)^b^
Early initiation of breastfeeding			
No	1920	56.6 (54.4, 59.1)	20.0 (17.6, 22.7)
Yes	1472	43.4 (40.9, 46.0)	20.2 (17.6, 23.1)
Exclusive breastfeeding			
No	447	51.4 (47.3, 55.6)	16.7 (12.4, 22.2)
Yes	422	48.6 (44.4, 52.8)	4.8 (3.0, 7.7)
Predominant breastfeeding			
No	782	89.9 (87.1, 92.2)	11.5 (9.0, 14.7)
Yes	88	10.1 (7.7, 12.9)	4.4 (1.5, 12.3)
Continued breastfeeding at 1 year			
No	59.5	10.4 (7.8, 13.6)	19.3 (8.1, 39.1)
Yes	515.2	89.6 (86.4, 92.2)	23.2 (19.9, 28.0)
Bottle feeding			
No	3267	96.3 (95.2, 97.2)	19.8 (17.8, 21.9)
Yes	125	3.7 (2.8, 4.7)	26.6 (17.8, 37.9)
Introduction of solid, semi-solid and soft foods at 6–8 months			
No	355	27.9 (245.0, 31.0)	11.87 (9.3, 15.0)
Yes	920	72.2 (69.0, 75.0)	25.82 (20.2, 32.4)

N* weighted total number of children aged 0–23 months for each IYCF indicator

^a^Prevalence represents the overall weighted proportion of children with diarrhoea for each level (‘Yes’, ‘No’) of IYCF indicators

^b^Prevalence represents the overall weighted proportion of children with diarrhoea for each level (‘Yes’, ‘No’) of infant and young child feeding indicators

When examining IYCF indicators in relation to the prevalence of diarrhoea, infants under 6 months of age who were exclusively and predominantly breastfed had a lower prevalence of diarrhoea compared to their counterparts who were not exclusively and predominantly breastfed (Table 2). In contrast, children aged 12–15 months who continued breastfeeding at 1 year had a higher prevalence of diarrhoea compared to their counterparts. Similarly, infants aged 6–8 months who were introduced to complementary foods had a higher prevalence of diarrhoea compared to those who received no complementary foods, that is, infants who were exclusively breastfed at 6–8 months. There was no difference in diarrhoea prevalence among children who were put to the breast within the first hour of birth and those who were not (Table 2).

Multivariable analyses revealed that infants aged 0–5 months who were exclusively breastfed were less likely to experience diarrhoea compared to those who were not exclusively breastfed [adjusted odds ratio (AOR) 0.31, 95% confidence interval (CI) 0.16–0.59, *P* < 0.001]. Infants who were predominantly breastfed were significantly less likely to experience diarrhoea compared to those who were not predominantly breastfed (AOR = 0.30, 95% CI 0.10–0.89, *P* = 0.031) [Table 3]. The analysis showed that infants who received solid, semi-solid and soft foods at age 6–8 months were approximately three times more likely to experience diarrhoea compared to their counterparts who received no complementary foods, that is, infants who were exclusively breastfed at 6–8 months (AOR = 2.91, 95% CI 1.99–0.89, *P* < 0.001). Children aged 12–15 months who continued breastfeeding at 1 year and those aged 0–23 months who were bottle-fed were more likely to experience diarrhoea, but these findings were not statistically significant (Table 3).

**Table 3:** Association between diarrhoea and infant and young child feeding (IYCF) indicators in Tanzania, TDHS 2010

IYCF indicators	Unadjusted OR (95% CI)	*P* value	^a^Adjusted OR (95% CI)	*P* value
Early initiation of breastfeeding				
No	1.00		1.00	
Yes	0.97 (0.79, 1.18)	0.749	1.00 (0.81, 1.25)	0.959
Exclusive breastfeeding				
No	1.00		1.00	
Yes	0.33 (0.18, 0.59)	< 0.001	0.31 (0.16, 0.59)	< 0.001
Predominant breastfeeding				
No	1.00		1.00	
Yes	0.29 (0.10, 0.83)	0.022	0.30 (0.10, 0.89)	0.031
Continued breastfeeding at 1 year				
No	1.00		1.00	
Yes	1.12 (0.42, 2.97)	0.820	1.24 (0.42, 3.64)	0.692
Bottle feeding				
No	1.00		1.00	
Yes	1.16 (0.73, 1.84)	0.509	1.36 (0.81, 2.29)	0.251
Introduction of solid, semi-solid and soft foods at 6–8 months				
No	1.00		1.00	
Yes	2.87 (1.98, 4.14)	< 0.001	2.91 (1.99, 4.27)	< 0.001

^a^Models adjusted for socioeconomic status (including maternal education, father’s education, employment, household wealth), health service (antenatal care visits), household factors (household location, type of toilet and source of drinking water) and individual factors (maternal age, child’s age, gender)

## Discussion

The present study found that the prevalence of diarrhoea was lower among infants whose mothers engaged in EBF and predominant breastfeeding (PBF) practices. The prevalence of diarrhoea was higher in children who continued breastfeeding at 1 year compared to those who had ceased breastfeeding at the same age. Children who were bottle-fed had a higher prevalence of diarrhoea compared to those who were not bottle-fed. Similarly, the prevalence of diarrhoea was higher in infants aged 6–8 months who received complementary foods compared to those who did not, that is, infants who were exclusively breastfed at 6–8 months. Our analyses revealed that EBF and PBF were protective against diarrhoeal illness in Tanzanian children aged 0–5 months, after adjustment for confounding variables. In contrast, infants aged 6–8 months who received complementary foods were approximately three times more likely to experience diarrhoea compared to those who received no complementary foods.

The study showed that the prevalence of diarrhoea was higher among infants who were not exclusively breastfed. Also, the likelihood of an infant aged under 6 months to experience diarrhoea was higher among those not exclusively breastfed compared to those who were exclusively breastfed. This finding is consistent with evidence from other developing countries, including Bangladesh [7], Vietnam [24] and Nigeria [10], where EBF was protective of diarrhoeal illness among infants of the same age, even after adjusting for poor sanitation and unsafe drinking water source [10, 24]. Similarly, a meta-analysis of 18 studies also reported the protective effects of EBF in reducing the burden of diarrhoeal illness [6]. Reasons for the protective effect of EBF on diarrhoea are based on the fact that EBF limits the infant’s exposure to contaminated liquids and foods, as well as the immunological activities of breast milk to protect the infant’s gastrointestinal tract from invading micro-organisms [5, 17]. Additionally, studies have reported that breast milk also stimulates the innate immune system [39, 40] and epigenetic programme of the infant, activities which are also essential for the prevention of infections [41].

Our study suggested that PBF was protective against diarrhoea among Tanzanian children, and this was consistent with previous studies from Nigeria [10], Bangladesh [7] and other SSA countries with high diarrhoea mortality [25]. However, studies from developing countries (such as Ethiopia and Burkina Faso) have shown that PBF increased the likelihood of the infant to experience diarrhoea [6, 24, 42–44] as parents or caregivers often insist that water—often ‘unimproved’ and a primary source of infection—induces suckling or quenches an infant’s thirst after breastfeeding [45–47]. Although PBF has the potential to increase the likelihood of the infant to experience diarrhoea because of the introduction of food-based fluids, Rajiv and colleagues have argued that promoting both EBF and PBF may be more beneficial than promoting EBF over PBF. The authors also noted that there is limited evidence to differentiate the benefits of EBF from those of PBF [11]. Our analysis underscores the fact that both EBF and PBF may have similar impacts on diarrhoea prevention among children aged 0–5 months in Tanzania. Nevertheless, efforts must be made to promote EBF over PBF as the possibilities of water or water-based food contamination in Tanzania are high because of limited access to improved drinking water source, improved sanitation and high-quality food storage facilities for mothers from low SES group and those living in urban slums or rural areas [48].

The study showed that continued breastfeeding at 1 year and bottle feeding were associated with a higher likelihood of the infant to experience diarrhoea, but the relationship was not statistically significant. Previous studies reported that continued breastfeeding at 1 year and bottle feeding were associated with an increased likelihood of the child to experience diarrhoea [10, 25]. Importantly, the impact of continued breastfeeding and bottle feeding on diarrhoea could be attributed to the introduction of contaminated complementary food/water or use of a contaminated bottle with a teat or nipple given the age intervals used in assessing those indicators correspond to a time when the infant has commenced complementary foods [10, 43, 49]. Similarly, the WHO considers bottle feeding as a breastfeeding indicator because of the association between bottle feeding and increased diarrhoeal morbidity and mortality [32, 37].

The present study suggested that timely introduction of solid, semi-solid, and soft foods was associated with an increased likelihood of the infant to experience diarrhoea. Prior studies have shown that the introduction of complementary foods in many developing countries (including Tanzania) predisposes the infant to experience diarrhoea because of the unsafe conditions in which the foods are handled, prepared and stored [14, 50], and this may be the case in our study. In Tanzania, maize is the most commonly used complementary foods for infants, but maize and maize-based foods have been shown to contain a high concentration of fumonisin—a diarrhoea causing mycotoxin [51–53]. A number of locally relevant approaches have been suggested to limit the infants’ exposure to high concentration of fumonisin-contaminated maize in Tanzania. These include maize sorting, dehulling of maize (removal of the hull from the seed) or limiting the daily intake of maize and/or replacing maize with other less fumonisin-contaminated cereal such as sorghum or finger millet [51]. However, there are significant challenges to the full implementation of those strategies. For example, resource constraint farmers are unwilling to sort out low-quality maize from their annual harvest [54, 55] or the household preference for the whole maize instead of the dehulled maize product [55]. Our finding implies that mothers should not only be encouraged to timely introduce complementary foods to their infants but also childhood nutrition intervention strategies must ensure that those foods are safe and nutritionally balanced, consistent with the WHO recommendation.

### Policy implications

Our study has policy and practical implications for public health experts, decision-makers and researchers as it further highlights the important role of IYCF practices in the context of diarrhoeal disease in a developing country. Accordingly, facility-based interventions such as Baby-Friendly Hospital Initiative (BFHI) to adequately promote and support optimal IYCF practices are essential to reduce diarrhoea burden. However, a report from Tanzania has shown that the BFHI is weak. That is, of the 3600 maternity service centres in the country, only 18 health facilities were certified baby friendly centres in 2015 [56], indicating that many health facilities and professionals may be unaware of the BFHI. This gap in the full implementation of the BFHI—shown to promote and support optimal IYCF behaviours [57–61]—suggests that more work is needed to improve breastfeeding behaviours of Tanzania mothers. Broadening the BFHI in Tanzania will not only improve EBF and reduce diarrhoea morbidity and mortality but could also increase household productivity due to a reduction in child sick days [5].

Furthermore, the World Breastfeeding Trends Initiative for Tanzania reported that the national-level policy framework and programme coordination to promote and support breastfeeding practices are ‘adequate’. Nevertheless, the full implementation of breastfeeding programmes at the health facility and community levels, as well as linkages between breastfeeding programmes at the national and subnational levels, are fragile [56]. Interventions to improve IYCF practices and reduce diarrhoea burden in Tanzania should include improvement in IYCF information for health professionals at the health system level, strengthening the routine postnatal care follow-up for mothers (such as subsidised or free maternal health care services) and scaling up community-based strategies (such as Baby-Friendly Community Initiatives, BFCI, and reinforcing linkages between facility and community-based programmes). Strengthening compliance with the Employment and Labour Relations Act 2004 and increasing domestic funding for IYCF programmes will also have impacts on breastfeeding practices [56, 62]. A detailed description of those facility- and community-based initiatives to improve IYCF behaviours in Tanzania has been noted elsewhere [56].

In 2016, the Government of the United Republic of Tanzania launched the National Multisectoral Nutrition Action Plan (NMNAP) for the years spanning 2016–2021, with the improvement of breastfeeding practices and the introduction of complementary foods being part of the rationale for this initiative [62]. Since the 1960s, Tanzania’s commitment to improving childhood nutrition has been long-lasting. However, the issue has been to translate the government commitment into evidence-based interventions that are appropriately financed, well-implemented and tracked [62]. To reduce the burden of diarrhoea associated with the introduction of complementary foods in Tanzania, a number of strategic approaches will be needed. These may include increased translation of government commitment to well-resourced and well-coordinated, measurable and culturally appropriate initiatives, and collaborative efforts between government agencies (such as health, agriculture and education) [62] and industry partners [63] to reduce the contamination associated with complementary foods. Additionally, studies that investigate the nutritional status of children may be warranted to inform childhood nutrition initiatives and policies in Tanzania.

### Study limitations and strengths

A number of methodological limitations should be considered. First, the study used cross-sectional data, and the establishment of a clear temporal association between IYCF and diarrhoea cannot be measured because the data were collected simultaneously. Second, the exposure and outcome variables were based on self-report, and this is a source of recall or measurement bias. The analysis may have overestimated or underestimated the association between IYCF practices and diarrhoea as the mother may have incorrectly reported the number of loose stools passed by the child. Nonetheless, our findings are consistent with previous evidence from developing countries on IYCF practices and diarrhoea [7, 10, 24]. Third, unmeasured confounding factors such as the impact of culture, and family structure and dynamics may be a limitation of the study. Fourth, data on the duration and severity of diarrhoea were unavailable, information that would have provided an in-depth understanding on the scope of protection from EBF and PBF and the pattern of diarrhoea in the context of IYCF practices. Finally, seasonal patterns exist for diarrhoea in SSA countries (including Tanzania) [64]. However, our study was limited to calendar years. Using monthly data would have provided better and detailed findings.

Despite the limitations, the study has strengths. First, we believe that the potential impact of selection bias is unlikely to affect the study findings given the nationally representative sample of participants and the subsequent response rate (96%). Second, the study used the TDHS data, which were collected with standardised questionnaires to ensure similarities across geographies in Tanzania. Finally, the study provides additional evidence on the impact of optimal IYCF practices on diarrhoea—the second most common cause of deaths among children under 5 years—to continue the advocacy for interventions that seek to promote child survival.

## Conclusion

The study found that diarrhoea prevalence was higher among children whose mothers practised suboptimal IYCF in Tanzania. EBF and PBF were protective against diarrhoeal illness, while the introduction of solid, semi-solid and soft foods to infants aged 6–8 months was associated with an increased onset of diarrhoea in Tanzanian children. Strengthening facility (e.g. BFHI and postnatal care follow-up)- and community (e.g. BFCI)-based linkages would help to improve IYCF and reduce under-five mortality from diarrhoea in Tanzanian children.

## Abbreviations

BFCI: Baby-Friendly Community Initiative
BFHI: Baby-Friendly Hospital Initiative
EBF: Exclusive breastfeeding
IYCF: Infant and young child feeding
PBF: Predominant breastfeeding
TDHS: Tanzania demographic and health survey
WHO: World Health Organization
